# Alterations in CD200-CD200R1 System during EAE Already Manifest at Presymptomatic Stages

**DOI:** 10.3389/fncel.2017.00129

**Published:** 2017-05-04

**Authors:** Tony Valente, Joan Serratosa, Unai Perpiñá, Josep Saura, Carme Solà

**Affiliations:** ^1^Department of Cerebral Ischemia and Neurodegeneration, Institut D’Investigacions Biomèdiques de Barcelona-Consejo Superior de Investigaciones Científicas (CSIC), Institut D’Investigacions Biomèdiques August-Pi i Sunyer (IDIBAPS)Barcelona, Spain; ^2^Biochemistry and Molecular Biology Unit, School of Medicine, Institut D’Investigacions Biomèdiques August-Pi i Sunyer (IDIBAPS), University of BarcelonaBarcelona, Spain

**Keywords:** CD200-CD200R1, EAE, multiple sclerosis, neuroinflammation, glial activation, neuron-glia communication, neurological disease, microglia

## Abstract

In the brain of patients with multiple sclerosis, activated microglia/macrophages appear in active lesions and in normal appearing white matter. However, whether they play a beneficial or a detrimental role in the development of the pathology remains a controversial issue. The production of pro-inflammatory molecules by chronically activated microglial cells is suggested to contribute to the progression of neurodegenerative processes in neurological disease. In the healthy brain, neurons control glial activation through several inhibitory mechanisms, such as the CD200-CD200R1 interaction. Therefore, we studied whether alterations in the CD200-CD200R1 system might underlie the neuroinflammation in an experimental autoimmune encephalomyelitis (EAE) model of multiple sclerosis. We determined the time course of CD200 and CD200R1 expression in the brain and spinal cord of an EAE mouse model from presymptomatic to late symptomatic stages. We also assessed the correlation with associated glial activation, inflammatory response and EAE severity. Alterations in CD200 and CD200R1 expression were mainly observed in spinal cord regions in the EAE model, mostly a decrease in CD200 and an increase in CD200R1 expression. A decrease in the expression of the mRNA encoding a full CD200 protein was detected before the onset of clinical signs, and remained thereafter. A decrease in CD200 protein expression was observed from the onset of clinical signs. By contrast, CD200R1 expression increased at EAE onset, when a glial reaction associated with the production of pro- and anti-inflammatory markers occurred, and continued to be elevated during the pathology. Moreover, the magnitude of the alterations correlated with severity of the EAE mainly in spinal cord. These results suggest that neuronal-microglial communication through CD200-CD200R1 interaction is compromised in EAE. The early decreases in CD200 expression in EAE suggest that this downregulation might also occur in the initial phases of multiple sclerosis, and that this early neuronal dysfunction might facilitate the development of neuroinflammation. The increased CD200R1 expression in the EAE model highlights the potential use of targeted agonist molecules as therapeutic tools to control neuroinflammation. In summary, the CD200-CD200R1 system is a potential therapeutic target in multiple sclerosis, and CD200R1 agonists are molecules that may be worth developing in this context.

## Introduction

Neuroinflammation plays a role in the pathogenesis and progression of multiple sclerosis (MS), involving immune cells from both the peripheral immune system and the central nervous system (CNS) (González et al., 2014). Because the modulation of neuroinflammation is considered a possible therapeutic strategy in MS, a thorough knowledge of the cellular and molecular mechanisms is necessary to identify candidate therapeutic targets. To what extent microglia, the main innate immune cells of the CNS, contribute to the development of MS is still a matter of debate (Bogie et al., 2014; Correale, 2014; Gertig and Hanisch, 2014).

Activated microglia/macrophages are not only found in active lesions but also in normal appearing white matter in the brains of MS patients, which suggests that microglia are involved from the initial stages of pathology (Allen et al., 2001; Zeis et al., 2008; van Horssen et al., 2012; Melief et al., 2013). Microglia are crucial in the maintenance of brain homeostasis, and activated microglia show a wide range of molecular and functional phenotypes, from the classically activated M1 phenotype, associated with a pro-inflammatory effect, to the alternatively activated M2 phenotype, associated with a beneficial effect (Napoli and Neumann, 2010; Gertig and Hanisch, 2014). The production of pro-inflammatory molecules by chronically activated microglial cells may contribute to the progress of neurodegenerative processes in neurological disease, and the inhibition of this response could be a therapeutic target. However, microglial functions also include promotion of neuronal survival, control of the inflammatory response, induction of phagocytosis of cellular debris and stimulation of tissue repair. Consequently, the precise role of microglia in the development of MS is still controversial. An optimal therapeutic approach targeting microglial cells could focus on modulating microglial activation to suppress their deleterious effects and promote their beneficial ones. In fact minocycline, a tetracycline antibiotic with anti-inflammatory properties, attenuates the development of pathology in the experimental autoimmune encephalomyelitis (EAE) animal model of MS through its action on microglia (Popovic et al., 2002). However, beneficial microglial responses are activated during the development of EAE, such as the induction of TREM-2 expression, which controls excess inflammation and stimulates phagocytosis of myelin debris (Takahashi et al., 2007).

Several pharmacological strategies in MS focus on the inhibition of the peripheral immune response, and their effect on microglial cells have been poorly investigated (Giunti et al., 2013). In fact, therapeutic strategies designed to modulate the innate immune response through an action on microglial cells are underexplored (Goldmann and Prinz, 2013). Potential candidates include the inhibitory mechanisms involved in the control of the microglial inflammatory response. Under physiological conditions, inhibitory mechanisms involved in neuron-glia communication participate in the control of the microglial inflammatory response, including CX3CL1-CXRCR1, CD172a-CD45, CD47-CD172, and CD200-CD200R1 ligand-receptor pairs (Tian et al., 2009; Jurgens and Johnson, 2012; Kierdorf and Prinz, 2013). In neurological diseases, the persistence of glial activation over time suggests a possible impairment of these inhibitory mechanisms. In the present study we focus on the CD200-CD200R1 system (Gorczynski, 2012; Walker and Lue, 2013). The CD200 molecule is a transmembrane glycoprotein mainly expressed by neurons and endothelial cells in the CNS, but that is also thought to be expressed by oligodendrocytes and astrocytes. Its receptor CD200R1 is mainly expressed by myeloid cells, like microglia or macrophages. CD200-CD200R1 interaction in myeloid cells results in the phosphorylation of a NPxY motif in the cytoplasmic domain of CD200R1 (Wright et al., 2000), which binds the phosphotyrosine-binding (PTB) domain in the adaptor molecules downstream of tyrosine kinase 1 (Dok1) and Dok2 upon tyrosine phosphorylation. This results in the recruitment and activation by phosphorylation of the Ras GTPase effector enzyme (RasGAP) (Zhang et al., 2004; Mihrshahi and Brown, 2010) and the inhibition of Ras and downstream MAPKs (mitogen-activated protein kinases) PI3K and ERK activation, leading to the inhibition of the production of inflammatory cytokines.

In both the mouse and the human brain, two CD200 isoforms are expressed by alternative mRNA splicing, a full form of the protein (CD200full) -the most abundant isoform- and a truncated form (CD200tr) (Borriello et al., 1998; Chen et al., 2008, 2010). CD200tr also binds to CD200R1, but does not activate the signal transduction pathway, acting as a physiological CD200 antagonist (Chen et al., 2008, 2010). In regard to CD200R1, a single mRNA variant has been described in the mouse brain (Wright et al., 2000), but four mRNA variants resulting from alternative splicing are described in the human brain (Vieites et al., 2003). While the mouse CD200R1 mRNA encodes a transmembrane protein, only two of the human CD200R1 mRNA variants encode transmembrane proteins whereas the other two encode shorter soluble proteins lacking the transmembrane and cytoplasmic domains. Little is known of the changes occurring in CD200 and CD200R1 expression, or in the mechanisms that regulate this expression under physiological and pathological conditions in the CNS, but it has been suggested that the CD200-CD200R1 system could be a candidate therapeutic target in MS (Koning et al., 2009b). The expression of molecules, such as CD200, involved in the control of the inflammatory response by microglia/macrophage, is modified in the *postmortem* brain tissue of patients with MS (Koning et al., 2007, 2009a). Decreased expression of CD200 and CD200R has been described in *postmortem* brain tissue of patients with Alzheimer’s disease (Walker et al., 2009). Several experimental approaches using the EAE model of MS have shown that reducing the CD200-CD200R1 interaction can aggravate the pathology (Hoek et al., 2000; Meuth et al., 2008), while facilitating CD200R1 activation can improve outcomes (Chitnis et al., 2007; Liu et al., 2010). Although studies in *postmortem* human tissue have allowed the characterization of the CD200-CD200R1 system at the final stages of the pathology, data are missing on the changes occurring in this system over time, and on their possible involvement in the development of MS. Finally, although manipulation of the CD200-CD200R1 interaction can modify the course of EAE, the extent to which the CD200-CD200R1 system is modified during EAE has not been studied to date.

We have recently shown that CD200R1 expression is inhibited *in vitro* in mouse reactive microglial cells, and that transcription factors involved in the control of the inflammatory response in these reactive microglia modulate CD200R1 expression (Dentesano et al., 2012, 2014). In the present study, we aimed to determine the dynamics of the CD200-CD200R1 system in EAE by looking at the changes in CD200 and CD200R1 expression in mouse CNS during the development of pathology in the context of associated glial activation and inflammation.

## Materials and Methods

### Animals

All animal experiments were performed in accordance with the Guidelines of the European Union Council (Directive 2010/63/EU) and Spanish Government (BOE 67/8509-12) and were approved by the Ethics and Scientific Committees of the Spanish National Research Council (CSIC) and the University of Barcelona. All protocols were registered at the *“Departament d’Agricultura, Ramaderia, Pesca, Alimentació i Medi Natural de la Generalitat de Catalunya”* (DARP 7065). Mice were maintained under regulated light and temperature conditions at the animal facilities of the Faculty of Medicine, University of Barcelona. All efforts were made to minimize animal suffering and discomfort and to reduce the number of animals used.

### EAE Model

The EAE model used female 6 to 8-week-old C57BL/6 mice (Harlan UK Ltd., Blackthom, UK), as previously described (Mannara et al., 2012). Briefly, mice were immunized under isoflurane anesthesia with a subcutaneous injection of an encephalitogenic emulsion containing 100 μg/mouse of myelin oligodendrocyte glycoprotein (MOG) peptide 35–55 (MOG_35-55_, Espikem, Italy) and 1 mg/mouse of H37R *Mycobacterium tuberculosis* (Difco, USA) in 200 μl of complete Freund’s adjuvant (CFA) (Sigma–Aldrich, St. Louis, MO, USA). These were then called MOG-EAE mice. Sham-treated mice were injected with a similar emulsion but without MOG_35-55_, and were used as controls. These were then called CFA mice. All mice were injected intraperitoneally with pertussis toxin from *Bordetella pertussis* (500 ng/mouse, Sigma–Aldrich) at 1 and 48 h after immunization. Body weight was checked daily from 7 days post-immunization (DPI). At the same time, clinical EAE symptoms (mobility loss and hind limb paralysis) were evaluated according to the following score: 0 = no symptoms; 0.5 = tail weakness; 1 = tail completely flaccid; 1.5 = low difficulty in righting; 2 = high difficulty in righting; 2.5 = unsteady gait and paraparesis (mild paralysis of one or two hind limbs); 3 = complete paralysis of one hind limb; 3.5 = complete paralysis of one hind limb and mild paralysis of the other hind limb; 4 = paraplegia (complete paralysis of two hind limbs) and incontinence; 4.5 = paraplegia and mild paralysis of one or two forelimbs; and 5 = moribund or dead.

For evaluation of clinical EAE symptoms, we used 11 CFA and 26 MOG-EAE mice in three independent experiments. MOG-EAE mice were killed at 28 DPI. In subsequent experiments, CFA and MOG-EAE mice were killed at the following points to determine cellular and molecular alterations in EAE: 9 DPI (presymptomatic phase), 14 DPI (symptomatic phase), 21 DPI (EAE peak) and 28 DPI (chronic phase). For mRNA and protein expression studies, 6 CFA and 19 MOG-EAE mice were considered, CFA mice were processed at 14 (*n* = 3) and 28 (*n* = 3) DPI, whereas MOG-EAE mice were processed at 9 (*n* = 4), 14 (*n* = 4), 21 (*n* = 5), and 28 (*n* = 6) DPI. Spinal cords and brains were carefully removed and longitudinally divided into equal left and right halves, to be processed for RNA or protein extraction, respectively. The spinal cord was dissected into cervical, thoracic, and lumbar regions, while the brain was dissected into mesencephalon plus diencephalon, the rhombencephalon, and the telencephalon regions. All samples were quickly frozen in dry ice. For histology, *in situ* hybridization and immunohistochemistry 5 CFA and 11 MOG-EAE mice were considered. MOG-EAE mice were perfused in 4% paraformaldehyde at 14 (*n* = 5) and 21 (*n* = 6) DPI as previously described (Mannara et al., 2012), while CFA mice were processed at 21 DPI (*n* = 5). Spinal cords and brains were carefully removed, post-fixed, and cryoprotected in a solution of 30% sucrose and 4% paraformaldehyde, and spinal cords were also divided into cervical, thoracic and lumbar regions. All samples were frozen in dry ice and 20 μm-thick cryostat coronal sections were obtained and stored at -20°C.

### Histology and Immunohistochemistry

Sequential sections from CFA and MOG-EAE mice (15 and 25 μm-thick) were used for histological analysis, as described previously (Mannara et al., 2012). Hematoxylin and eosin staining was performed to evaluate the general status of the tissue and the presence of cell infiltration. Luxol fast blue method was used for staining myelin projections. Mouse sections were processed for immunohistochemistry as described previously (Valente et al., 2012). The primary and secondary antibodies used are shown in **Table 1**. Microscopy images were obtained with an Eclipse 1000 Nikon microscope (Nikon, Tokyo, Japan) and a digital camera (Olympus DP72, Tokyo, Japan).

**Table 1:** Antibodies used in western blot assays and immunocytochemistry.

Primary antibody	Species	Company	Dilution	Secondary antibody	Dilution	Company
**Western blot**						
β-Actin	Mouse	Sigma–Aldrich	1/40000	Goat anti-mouse HRP	1/5000	Santa Cruz Biotech.
β-Tubulin	Mouse	Sigma-Aldrich	1/1000	Goat anti-mouse HRP	1/5000	Santa Cruz Biotech.
CD200	Goat	R&D	1/1000	Mouse anti-goat HRP	1/2000	Sigma–Aldrich
CD200R1	Goat	Santa Cruz Biotech.	1/300	Mouse anti-goat HRP	1/2000	Sigma–Aldrich
**Immunohistochemistry**						
CD200	Goat	R&D	1/50	Alexa 488 donkey anti-goat	1/1000	Invitrogen
CD200	Rabbit	BIOSS	1/50	Alexa 488 donkey anti-rabbit	1/1000	Invitrogen
CD200R1	Goat	R&D	1/500	Alexa 488 donkey anti-goat	1/1000	Invitrogen
GFAP	Rabbit	DAKO	1/1000	Alexa 546 donkey anti-rabbit Biotinylated goat anti-rabbit	1/1000 1/500	Invitrogen Vector
Iba1	Rabbit	Wako	1/1000	Alexa 546 donkey anti-rabbit Biotinylated goat anti-rabbit	1/1000 1/500	Invitrogen Vector
MBP	Rat	Millipore	1/1000	Biotinylated goat anti-rat	1/1000	Invitrogen
NeuN	Mouse	Millipore	1/500	Alexa 546 donkey anti-rabbit	1/1000	Invitrogen

*CD200R1, CD200 receptor 1; GFAP, glial fibrillary acidic protein; Iba1, ionized calcium binding adaptor molecule 1; MBP, myelin basic protein; NeuN, Neuronal nuclei.*

*Companies: Sigma–Aldrich, St. Louis, MO, USA; R&D Systems Inc., Minneapolis, MN, USA; Santa Cruz Biotechnology, Inc., Dallas, Texas, USA; BD Pharmingen: BD Bioscencies, San José, CA, USA; Abcam plc, Cambridge, United Kingdom; Bioss antibodies, Woburn Massachusetts, USA; Dako, Carpinteria, CA, USA; Wako Pure Chemical Industries Ltd, Tokyo, Japan; Millipore, Bedford, MA, USA; Invitrogen Molecular Probes, Eugene, OR, USA; Vector Laboratories Inc., Burlingame, CA, USA.*

### *In Situ* Hybridization

Digoxigenin-d-UTP (Boehringer-Mannheim, Mannheim, Germany)-labeled antisense and sense riboprobes were obtained using mouse CD200 cDNA (a kind gift from Prof. R. Gorczynski, Toronto General Research Institute, Canada) and commercial CD200R1 cDNA (OriGene, Rockville, MD, USA). *In situ* hybridization was performed on mouse brain and spinal cord sections, as described previously (Valente et al., 2005).

### Quantitative Real-Time Polymerase Chain Reaction

Total RNA was isolated from frozen tissue samples, using the Trizol method (Tri^®^Reagent, Sigma–Aldrich). One microgram of RNA was reverse transcribed with random primers using Transcriptor Reverse Transcriptase (Roche Diagnostics Scheiwz AG, Rotkreuz, Switzerland). Then, cDNA was diluted 1/30 to perform quantitative real-time polymerase chain reaction (qRT-PCR) with IQ SYBRGREEN SuperMix (Bio-Rad Laboratories, Hercules, CA, USA) as previously described (Valente et al., 2012). The primers (Integrated DNA Technologies, Leuven, Belgium) used to amplify mouse mRNA are shown in **Table 2**. Relative gene expression was calculated by the comparative Ct or ΔΔCt method (Livak and Schmittgen, 2001) using CFX 2.1 software (Bio-Rad Laboratories).

**Table 2:** Primers used for qRT-PCR.

Murine			
**Target mRNA**	**Accession number**	**Forward primer (5′→3′)**	**Reverse primer (5′→3′)**

Arg1	NM_007482.3	TTGCGAGACGTAGACCCTGG	CAAAGCTCAGGTGAATCGGC
Cd200full	NM_010818.3	GGGCATGGCAGCAGTAGCG	TGTGCAGCGCCTTTCTTTC
Cd200tr	#	GATGGGCAGTCTGTGGAAGTG	GAGAACATCGTAAGGATGCAGTTG
Cd200r1	NM_021325.3	AGGAGGATGAAATGCAGCCTTA	TGCCTCCACCTTAGTCACAGTATC
COX2	NM_011198.3	TGCAGAATTGAAAGCCCTCT	CCCCAAAGATAGCATCTGGA
Gfap	NM_001131020.1	AAGGTCCGCTTCCTGGAA	GGCTCGAAGCTGGTTCAGTT
Iba1	NM_019467.2	GAAGCGAATGCTGGAGAAAC	AAGATGGCAGATCTCTTGCC
Il1b	NM_008361.3	TGGTGTGTGACGTTCCCATTA	CAGCACGAGGCTTTTTTGTTG
Il10	NM_010548.2	TGAATTCCCTGGGTGAGAAG	ACACCTTGGTCTTGGAGCTT
Mrc1	NM_008625.2	TCTTTTACGAGAAGTTGGGGTCAG	ATCATTCCGTTCACCAGAGGG
Nos2	NM_010927.3	GGCAGCCTGTGAGACCTTTG	GCATTGGAAGTGAAGCGTTTC
Socs3	NM_007707.3	GGGTGGCAAAGAAAAGGAG	GTTGAGCGTCAAGACCCAGT
Tgfb	NM_011577.1	TGCGCTTGCAGAGATTAAAA	AGCCCTGTATTCCGTCTCCT
Tnfa	NM_013693.2	TGATCCGCGACGTGGAA	ACCGCCTGGAGTTCTGGAA
Rn18s^∗^	NR_003278.3	GTAACCCGTTGAACCCCATT	CCATCCAATCGGTAGTAGCG

*#Chen et al., 2010; ^∗^reference gene; Arg1, arginase1; CD200full, full-length CD200; CD200tr, truncated CD200; CD200R1, CD200 receptor 1; CD200R1-Large, CD200R1 mRNA variants 1 and 4 (large mRNA variants); CD200R1-Short, CD200R1 mRNA variants 2 and 3 (short mRNA variants); CD200R1-V1, CD200R1 mRNA variant 1; CD200R1-V2, CD200R1 mRNA variant 2; CD200R1-V3, CD200R1 mRNA variant 3; CD200R1-V4, CD200R1 mRNA variant 4; COX2, cyclooxygenase-2; Gfap, glial fibrillary acidic protein; Iba1, ionized calcium binding adaptor molecule 1; Il1b, interleukin 1 beta; Il10, interleukin 10; Mrc1, mannose receptor 1; Nos2, nitric oxide synthase 2; Socs3, suppressor of cytokine signaling 3; Tgfb, transforming growth factor beta; Tnfa, tumor necrosis factor alpha; Rn18s, 18S ribosomal RNA; RPS18, ribosomal protein S18.*

### Western Blot

Mouse total protein extracts were obtained as previously described (Valente et al., 2012). Protein quantification was determined by Bradford assay (Bio-Rad Laboratories). Western blot analysis of 30 μg extracts of total protein was performed using appropriate primary and secondary antibodies (**Table 1**), as previously described (Valente et al., 2012). Membranes were developed with ECL-Plus (Amersham GE Healthcare Life Sciences Europe GmbH, Barcelona, Spain) and images were obtained using a VersaDoc System camera (Bio-Rad Laboratories). Data were expressed as the ratio between the band intensity of the protein of interest and that of the loading control (β-actin).

### Data Presentation and Statistical Analysis

Normality of data was determined by D’Agostino and Pearson omnibus normality test. Statistical analyses were performed using one-way analysis of variance (ANOVA) and the Dunnett *post hoc* test. The Spearman correlation coefficient (*r*) was calculated to measure the linear correlation between the clinical EAE score and mRNA expression. Statistical analyses were performed using GraphPad Prism 4.02 (GraphPad Software, Inc., La Jolla, CA, USA). All results are presented as mean ± SEM values, unless otherwise stated. Values of *p* < 0.05 were considered statistically significant.

## Results

### EAE Model and Glial Activation

**FIGURE 1:**
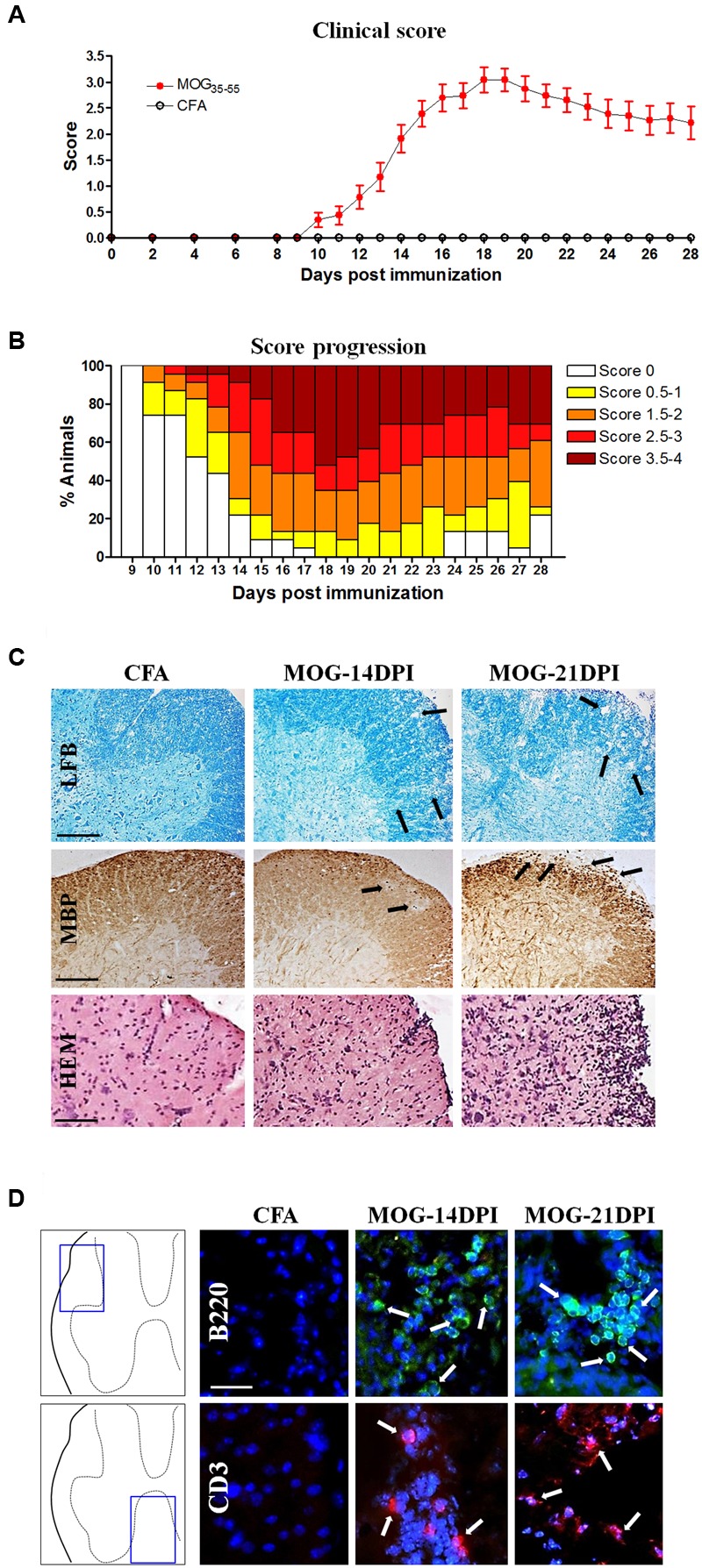
**The experimental autoimmune encephalomyelitis (EAE) model in C57BL/6J mice. (A)** The EAE score during 28 days post-immunization (DPI) (CFA mice, *n* = 11; MOG-EAE mice, *n* = 26). EAE symptoms appeared at 10 DPI and the peak of EAE score was reached at 18 DPI. **(B)** Progression of incidence and clinical score in MOG-EAE mice. **(C)** EAE histological features in the lumbar spinal cord: some areas of demyelination (arrows in Luxol fast blue –LFB- and myelin basic protein –MBP- labeling) were observed at 14 and 21 DPI in MOG-EAE mice when compared with CFA mice (black arrows); infiltrates were detected by hematoxylin and eosin staining (HEM). Scale bars: 250 μm in Luxol fast blue and MBP images, and 200 μm in HEM staining. **(D)** Lymphocyte infiltrates are evident in the lumbar spinal cord (arrows). B220-positive B-cell (green) and CD3-positive T-cell (red) infiltrates were found at 14 and 21 DPI. Scale bar: 100 μm.

To determine the kinetics of CD200-CD200R axis dysregulation during EAE, we first characterized EAE in this study, because this is known to be dependent on the facility/environment. MOG-EAE mice showed the first clinical signs of EAE at 10 DPI, with a mean onset at 12.9 ± 0.5 DPI (**Figure 1A**). The incidence of EAE was 26.1, 78.3, and 100%, at 10, 14, and 18 DPI, respectively (**Figure 1B**). At 18 DPI, 13.0% of the animals showed limp tails (score 0.5–1.0), 21.7% showed dysfunctional motor coordination (score 1.5–2.0) and 65.2% showed mild-to-moderate paraparesis (score 2.5–4.0). The EAE clinical data correlated with the demyelination observed in the spinal cord of MOG-EAE mice at 14 and 21 DPI. Thus, some small patches of demyelination were observed in the white matter of the dorsolateral spinal cord at 14 DPI, which became more evident at 21 DPI (**Figure 1C**). Infiltrating cells were detected by hematoxylin and eosin staining, which were evaluated by specific lymphocyte markers in the white matter of MOG-EAE mice, and were mainly localized around the marginal zone of demyelinated areas (**Figure 1C**), close to the spinal cord surface. Some cells were observed at 14 and 21 DPI showing B- and T-lymphocyte markers (B220 and CD3) (**Figure 1D**).

**FIGURE 2:**
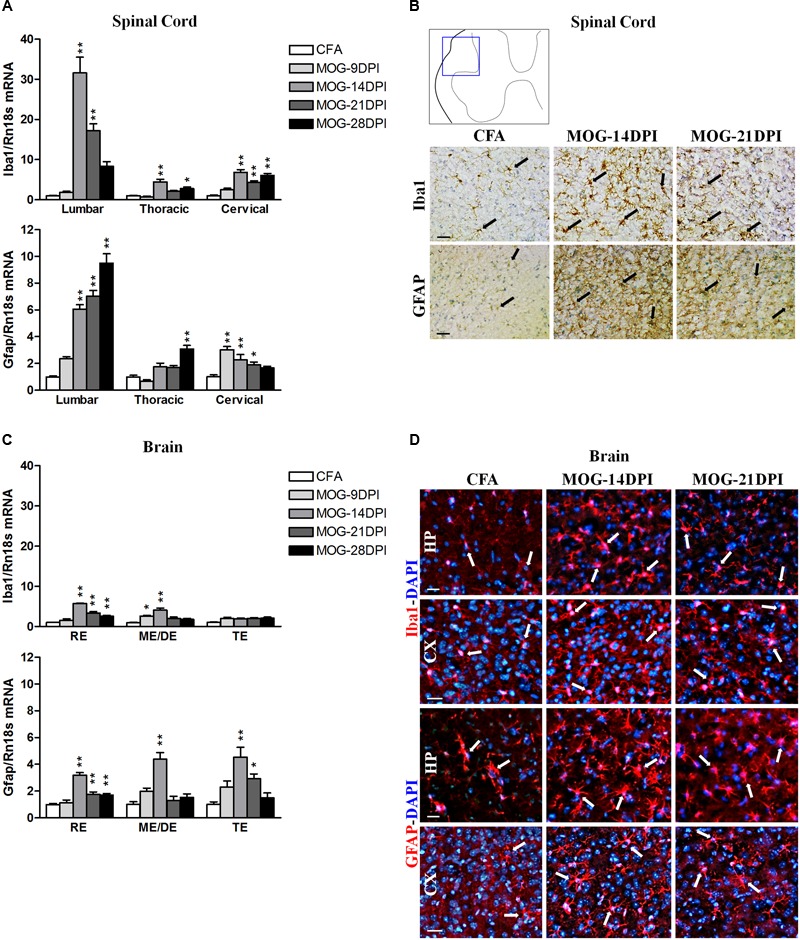
**Glial activation in the CNS of MOG-EAE mice. (A)** The mRNA expressions of Iba1 (microglial marker) and Gfap (astrocyte marker) were evaluated in spinal cord regions by qRT-PCR during EAE, using Rn18s as the housekeeping gene. **(B)** Iba1 and GFAP immunoreactivity (arrows) in CFA and MOG-EAE mice spinal cord at 14 and 21 DPI. Results from lumbar spinal cord, as representative of all spinal cord areas studied, are shown. **(C)** Iba1 and Gfap mRNA expressions in brain areas of CFA and MOG-EAE mice during EAE progression. **(D)** Immunocytochemistry showing Iba-1 and GFAP positive cells (red) in the CA1 field of the hippocampus (HP) and the temporal cortex (CX). Cells were counterstained with DAPI (blue). Bars represent the means ± SEM of 4–6 animals. ^∗^*p* < 0.05, ^∗∗^*p* < 0.01 vs. respective CFA; one-way ANOVA and Dunnett post-test. RE, rhombencephalon; ME/DE, mesencephalon/diencephalon; TE, telencephalon. Scale bars: 100 μm.

Regarding glial cells, we detected significant increases in the mRNA expressions of both the microglia/macrophage marker Iba1 and the astroglial marker GFAP in spinal cord regions of MOG-EAE mice at different post-immunization times (**Figure 2A**), albeit with some exceptions. Larger increases were observed in the lumbar spinal cord. Curiously, in the presymptomatic EAE phase (9 DPI), Gfap mRNA upregulation was observed in the cervical region. In MOG-EAE mice, strong Iba1 immunostaining was observed in spinal cord white matter at 14 and 21 DPI, suggesting microglia/macrophage activation. In addition, an increase in GFAP immunostaining was observed at 14 and 21 DPI in spinal cord white matter, which suggested astroglial activation. **Figure 2B** shows images from lumbar spinal cord as representative region showing the most apparent glial reactivity in the spinal cord. We also determined Iba1 and Gfap mRNA expression in all brain regions (**Figure 2C**) of MOG-EAE mice at different post-immunization times. Interestingly, upregulation of Iba1 mRNA could be observed in the presymptomatic phase in mesencephalon/diencephalon (**Figure 2C**). Microglial/macrophage activation was confirmed by Iba1 immunostaining in coronal brain sections (**Figure 2D**), where reactive microglia/macrophages were detected in the hippocampus at 14 and 21 DPI. GFAP-labeled reactive astrocytes were also observed in hippocampal and cortical areas (**Figure 2D**).

### Time Course of CD200 Expression in EAE

**FIGURE 3:**
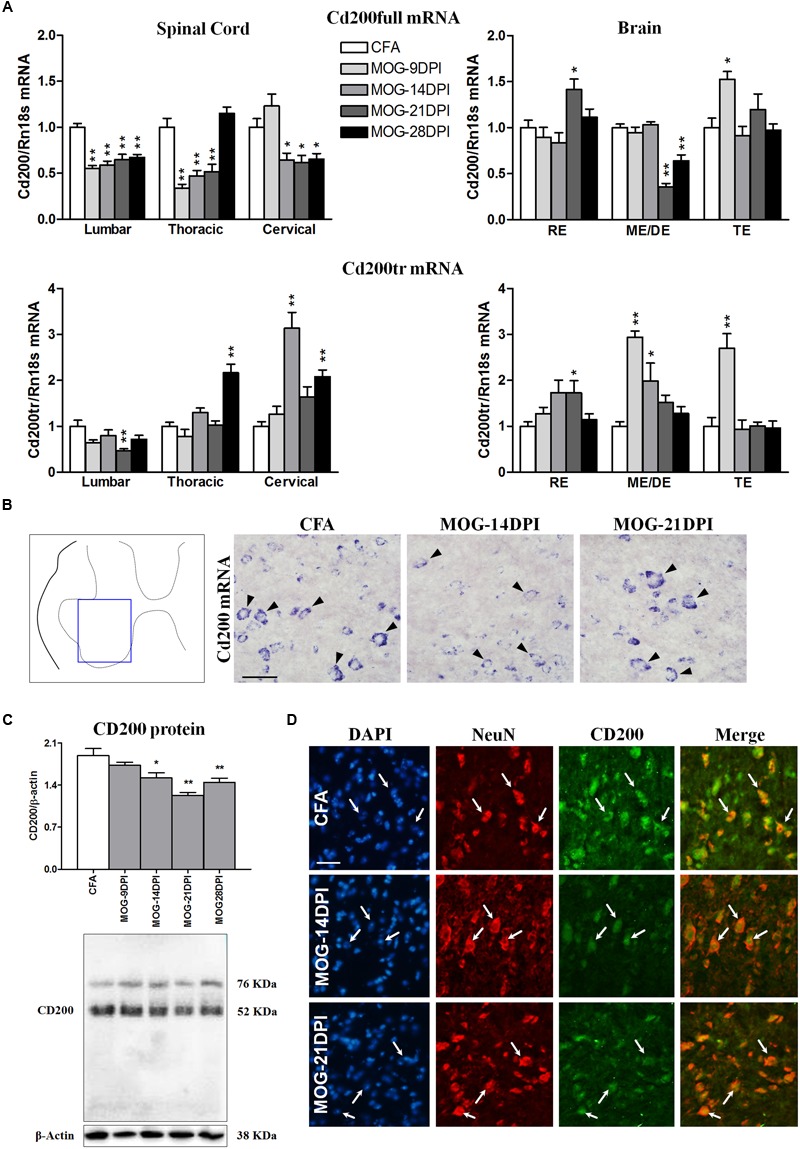
**Time course of CD200 expression in EAE. (A)** Full-length Cd200 (Cd200full) and truncated Cd200 (Cd200tr) mRNAs were evaluated in spinal cords and brains by qRT-PCR during EAE, using Rn18s as the housekeeping gene. Bars are the means ± SEM of 4–6 animals. ^∗^*p* < 0.05, ^∗∗^*p* < 0.01 vs. respective CFA; one-way ANOVA and Dunnett post-test. **(B)** *In situ* hybridization of Cd200full mRNA in the spinal cord showing Cd200full-positive cells (arrowheads) in the ventral gray matter. **(C)** Expression of CD200 protein in the spinal cord by western blot using β-actin as the loading control. Bars are the means ± SEM of 4–6 animals. ^∗^*p* < 0.05, ^∗∗^*p* < 0.01 vs. respective CFA; one-way ANOVA and Dunnett post-test. A representative western blot is shown. **(D)** Double immunofluorescence of CD200 (green) and NeuN (red) in the gray matter of the spinal cord (arrows). Cells were counterstained with DAPI (blue). Results from lumbar spinal cord, as representative of all spinal cord areas studied, are shown in **(B–D)**. RE, rhombencephalon; ME/DE, mesencephalon/diencephalon; TE, telencephalon. Scale bars: 100 μm.

We studied whether the immune reaction associated to MOG injection produced changes in the expression of the inhibitory immune receptor, CD200R1, and its ligand, CD200, in the CNS. To this end, we determined their mRNAs and/or protein expression in the spinal cords and brains of CFA and MOG-EAE mice after several DPI. In MOG-EAE mice, downregulation of Cd200full mRNA expression was observed in the presymptomatic phase of EAE (9 DPI) in lumbar and thoracic regions, but was observed in all spinal cord regions during symptomatic phases, except at 28 DPI in the thoracic region (**Figure 3A**). The magnitude of changes was similar among the spinal cord regions. Less-marked alterations were observed in the brain than in the spinal cord, with a decrease in Cd200full mRNA expression in the mesencephalon/diencephalon, and punctual increases in the rhombencephalon and telencephalon (**Figure 3A**). This contrasted with Cd200tr mRNA expression, which was increased in the thoracic and cervical spinal cord in the symptomatic phase and in the mesencephalon/diencephalon and telencephalon in the presymptomatic phase of MOG-EAE mice (**Figure 3A**). Cd200full mRNA expression negatively correlated with EAE clinical score in thoracic and cervical spinal cord areas, while there was a positive correlation between Cd200tr mRNA expression and the EAE clinical score in the cervical spinal cord (**Table 3**). By *in situ* hybridization, we observed cells positive for Cd200full mRNA with neuronal morphology only in the ventral part of the gray matter of the spinal cords of CFA mice, where the somas of motor neurons are localized (**Figure 3B**). However, cellular Cd200full mRNA expression was noticeably decreased in MOG-EAE mice at 14 and 21 DPI (**Figure 3B**). To determine whether CD200 transcript modulation during EAE translated to protein changes, we analyzed spinal cord CD200 expression by western blot. The anti-CD200 antibody used recognized amino acids 31–232 in the CD200 protein, and consequently the full-length and truncated isoforms (45–48 and 35–40 kDa, respectively) (Gorczynski, 2002). A main dense band was observed at approximately 52 kDa. From 14 DPI, there was a significant decrease in CD200 protein in the MOG-EAE mice (**Figure 3C**). The decrease observed by western blot was confirmed by CD200 immunofluorescence on spinal cord sections (**Figure 3D**), where CD200 was found to co-localize with the neuronal marker NeuN in the ventral part of the gray matter of the spinal cord.

**Table 3:** Correlation between EAE severity and Cd200full, Cd200tr or Cd200r1 mRNA expression in symptomatic MOG-EAE mice.

	Spinal cord			BRAIN		
Target mRNA	Lumbar (*n* = 15)	Thoracic (*n* = 19)	Cervical (*n* = 19)	RE (*n* = 18)	ME/DE (*n* = 14)	TE (*n* = 20)
	**Spearman *r***	**Spearman *r***	**Spearman *r***	**Spearman *r***	**Spearman *r***	**Spearman *r***

Cd200full	-0.4312	-0.7037^∗∗∗^	-0.6862^∗∗^	-0.0042	-0.0203	-0.1676
Cd200tr	-0.3424	-0.0803	+0.7212^∗∗∗^	+0.4648	+0.5252^∗∗^	-0.1130
Cd200r1	+0.7501^∗∗^	+0.7154^∗∗∗^	+0.8161 ^∗∗∗^	+0.3961	+0.6120^∗^	-0.0008

*^∗^*p* < 0.05, ^∗∗^*p* < 0.01, ^∗∗∗^*p* < 0.001.*

### Time Course of CD200R1 Expression in EAE

**FIGURE 4:**
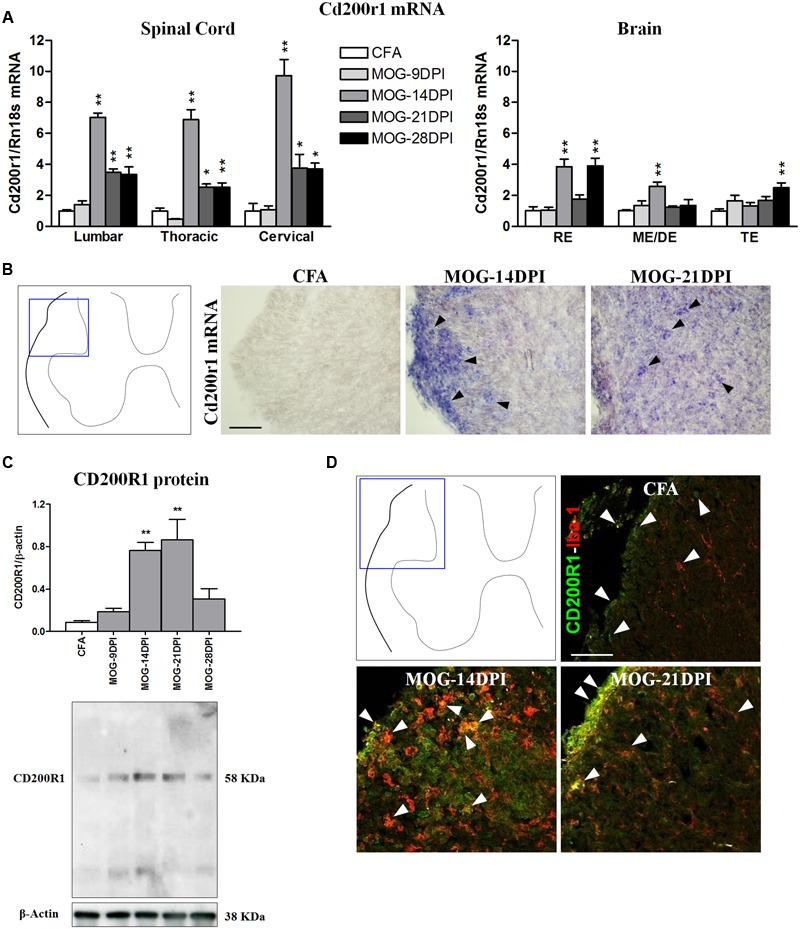
**Time course of CD200R1 expression in EAE. (A)** Cd200r1 mRNA was evaluated in the spinal cord and brain by qRT-PCR during EAE, using Rn18s as the housekeeping gene. Bars are the means ± SEM of 4–6 animals. ^∗^*p* < 0.05, ^∗∗^*p* < 0.01 vs. respective CFA; one-way ANOVA and Dunnett post-test. **(B)** *In situ* hybridization of Cd200r1 mRNA in the spinal cord. Cd200r1-positive cells (arrowheads) were detected in MOG-EAE mice. **(C)** CD200R1 protein expression in the spinal cord by western blot using β-actin as the loading control. Bars are the means ± SEM of 4–6 animals. ^∗∗^*p* < 0.01 vs. respective CFA; one-way ANOVA and Dunnett post-test. A representative western blot is shown. **(D)** Double immunofluorescence of CD200R1 (green) and Iba1 (red) in white matter of the spinal cord (arrowheads). Results from lumbar spinal cord, as representative of all spinal cord areas studied, are shown in **(B–D)**. RE, rhombencephalon; ME/DE, mesencephalon/diencephalon; TE, telencephalon. Scale bars: 100 μm.

In MOG-EAE mice, Cd200r1 mRNA expression was strongly upregulated in all the spinal cord regions at all symptomatic phases, showing a clear peak at 14 DPI (**Figure 4A**). Induction of Cd200r1 mRNA was also observed in the brain, but to a lesser extent than in the spinal cord. In addition, the EAE clinical score was positively correlated with Cd200r1 mRNA expression, mainly in spinal cord areas (**Table 3**). This increased expression was corroborated by *in situ* hybridization using spinal cord sections (**Figure 4B**). Thus, although no Cd200r1 positive cells were detected in the CFA mice, they were clearly observed in MOG-EAE mice at both 14 and 21 DPI. Cells positive for Cd200r1 mRNA were mainly localized to the white matter, mostly in areas of demyelination and infiltration in the lateral part of the dorsal spinal cord. Nevertheless, scattered CD200R1 and Iba1 positive cells were also observed in more distal areas. Western blotting of spinal cord total protein showed an increase in CD200R1 in protein extracts of MOG-EAE mice at 14 and 21 DPI (**Figure 4C**). The cellular localization of CD200R1 protein was then analyzed by immunohistochemistry (**Figure 4D**). Few CD200R1-positive cells were observed in the spinal cords of CFA mice, and this number increased markedly in MOG-EAE mice in the areas where cells positive for Cd200r1-mRNA-were found. In most cells, CD200R1 co-localized with the microglial/macrophage marker Iba1.

### Inflammatory Response in EAE

**FIGURE 5:**
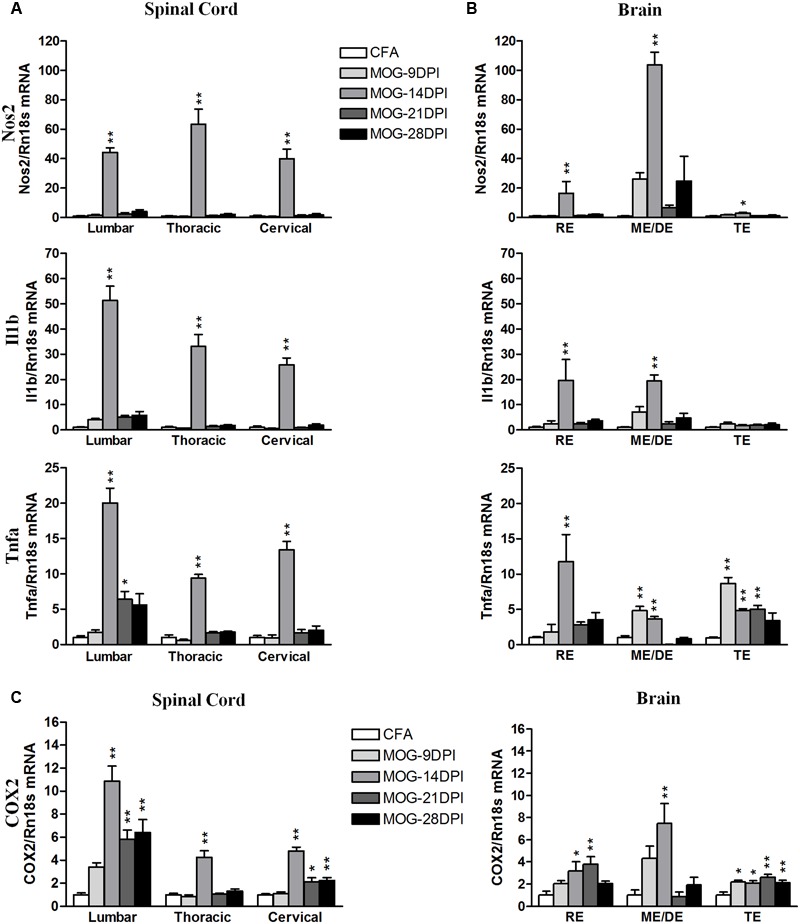
**Time-course expression of pro-inflammatory genes in EAE.** The mRNAs of the pro-inflammatory genes Nos2, Il1b, and Tnfa were evaluated in the spinal cord **(A)** and brain **(B)** by qRT-PCR during EAE. **(C)** The mRNA expression of COX2, pro- and anti-inflammatory gene, was evaluated in the spinal cord and brain by qRT-PCR. Rn18s was used as the housekeeping gene. Bars are the means ± SEM of 4–6 animals. ^∗^*p* < 0.05, ^∗∗^*p* < 0.01 vs. respective CFA; one-way ANOVA and Dunnett post-test. RE, rhombencephalon; ME/DE, mesencephalon/diencephalon; TE, telencephalon.

As the CD200-CD200R1 interaction is involved in the control of the inflammatory response in microglial cells, we analyzed the inflammatory context in which changes in CD200 and CD200R1 expression were observed. We studied the time course of pro- and anti-inflammatory molecule expression during the development of EAE using qRT-PCR. For classical pro-inflammatory M1 markers, we analyzed Nos2, Il1b, and Tnfa (**Figures 5A,B**). In the spinal cords of MOG-EAE mice, the respective genes showed mRNA expression profiles characterized by markedly peaked expression at 14 DPI (**Figure 5A**). In the brain, an increase in Nos2 mRNA was also observed at 14 DPI, particularly in the mesencephalon/diencephalon, while Il1b mRNA was significantly upregulated in the rhombencephalon and mesencephalon/diencephalon (**Figure 5B**). By contrast, Tnfa mRNA upregulation was observed in all brain areas at several times after immunization (**Figure 5B**), and notably in both the mesencephalon/diencephalon and telencephalon in the presymptomatic phase (9 DPI). We also analyzed COX2 mRNA expression, an M1/M2 marker, and found its levels significantly upregulated in all spinal cord areas during the symptomatic phases in MOG-EAE mice (i.e., 14–28 DPI). A significant increase in COX2 mRNA expression was also observed in all brain areas, but to a lesser extent than in the spinal cord (**Figure 5C**). In addition, COX2 mRNA upregulation was significantly increased in the telencephalon during the presymptomatic phase of EAE (9 DPI) (**Figure 5C**).

**FIGURE 6:**
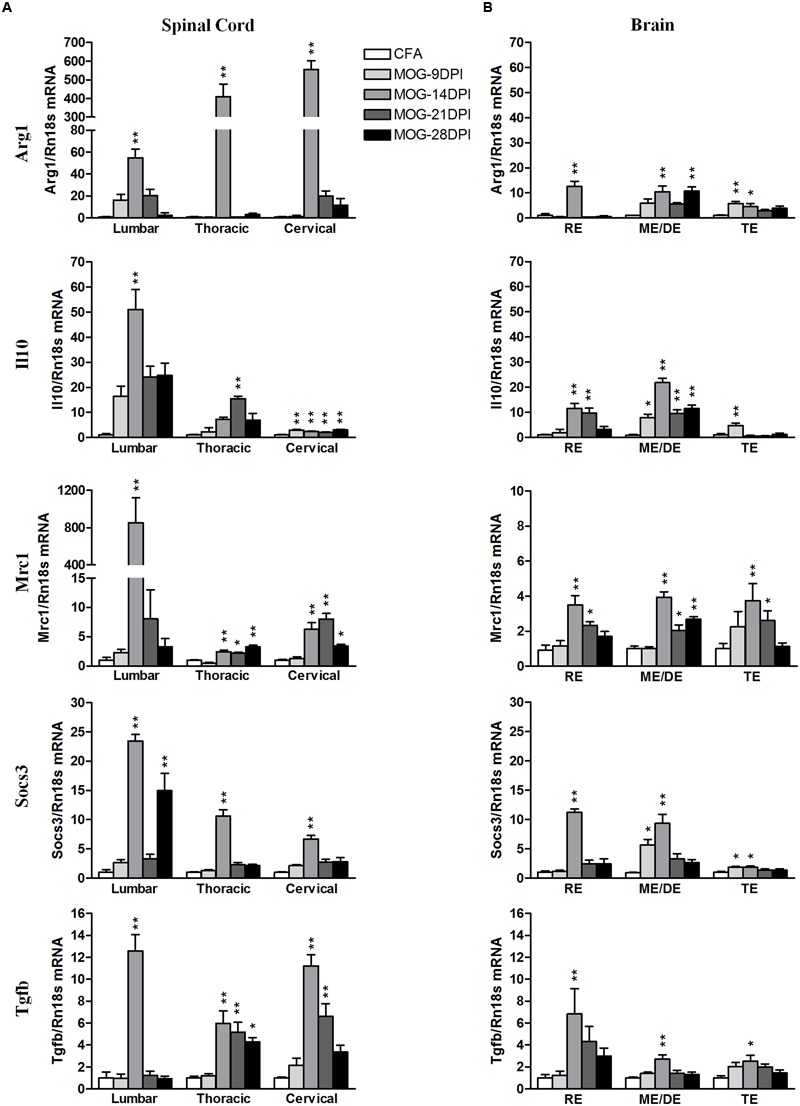
**Time-course expression of anti-inflammatory genes in EAE.** The mRNAs of anti-inflammatory genes Arg1, Il10, Mrc1, Socs3, and Tgfb were evaluated in the spinal cord **(A)** and brain **(B)** by qRT-PCR during EAE. Rn18s was used as the housekeeping gene. Bars are the means ± SEM of 4–6 animals. ^∗^*p* < 0.05, ^∗∗^*p* < 0.01 vs. respective CFA; one-way ANOVA and Dunnett post-test. RE, rhombencephalon; ME/DE, mesencephalon/diencephalon; TE, telencephalon.

We analyzed the following M2 genes as anti-inflammatory M2 markers: Arg1, Il10, Mrc1, Socs3, and Tgfb (**Figure 6**). Arg1 mRNA was strongly upregulated in spinal cord regions at 14 DPI (**Figure 6A**), while more moderate increases were found in all brain areas (**Figure 6B**). However, we also observed Arg1 mRNA induction during the presymptomatic phase in the telencephalon. For Il10 mRNA expression, we observed expression induction in spinal cord and brain regions between 14 and 28 DPI (**Figure 6**). The largest increase was observed in lumbar spinal cord at 14 DPI. In addition, Il10 mRNA expression was observed in the cervical spinal cord, mesencephalon/diencephalon, and telencephalon during the presymptomatic phase. Mrc1 mRNA expression was strongly upregulated in lumbar spinal cord, but significant increases were observed in the other spinal cord areas and brain tissue at several symptomatic phases (**Figure 6**). Socs 3 expression was mainly upregulated by 14 DPI in spinal cord and brain areas, but also during the presymptomatic phase in the brain (**Figure 6B**). Tgfb mRNA expression was significantly upregulated in the spinal cord during the symptomatic phase, but only became significant in the brain at 14 DPI (**Figure 6**).

Finally, we analyzed the correlation between EAE severity and pro- and anti-inflammatory mRNA expression in symptomatic MOG-EAE mice, which showed that the EAE clinical score was positively correlated with the expression levels of both pro- and anti-inflammatory genes in spinal cord, especially in the lumbar region, and brain areas, mainly in the rhombencephalon and mesencephalon/diencephalon (**Table 4**).

**Table 4:** Correlation between EAE severity and pro- and anti-inflammatory mRNA expression in symptomatic MOG-EAE mice.

	Spinal cord			Brain			
Target mRNA	Lumbar (*n* = 15)	Thoracic (*n* = 19)	Cervical (*n* = 19)	RE (*n* = 19)	ME/DE (*n* = 15)	TE (*n* = 20)
	**Spearman r**	**Spearman r**	**Spearman r**	**Spearman r**	**Spearman r**	**Spearman r**

**Pro-inflammatory**						
Nos2	+0,7519^∗∗^	+0,3713	+0,3207	+0,4562^∗^	+0,7929^∗∗∗^	+0,3781
Il1b	+0,6002^∗^	+0,4715^∗^	+0,1238	+0,3153	+0,6340^∗^	+0,2534
Tnfa	+0,6522^∗∗^	+0,4652^∗∗^	+0,5802	+0,6612^∗∗^	+0,2138	+0,5035^∗^
**Pro-/Anti-inflammatory**						
COX2	+0,7120^∗∗^	+0,2331	+0,3595	+0,0447^∗^	+0,5262^∗^	+0,6190^∗∗^
**Anti-inflammatory**						
Arg1	+0,3352	+0,5050^∗^	+0,3514	+0,4742^∗^	+0,3764	+0,4023
Il10	+0,8352^∗∗∗^	+0,4473	+0,1915	+0,4968^∗^	+0,5974^∗^	-0,1840
Mrc1	+0,7465^∗∗^	+0,2755	+0,5664^∗^	+0,4209^∗^	+0,6556^∗^	+0,6635^∗∗^
Socs3	+0,7193^∗∗^	+0,7007^∗∗^	+0,4175	+0,4869^∗^	+0,8642^∗∗∗^	+0,3547
Tgfb	+0,0857	+0,5641^∗^	+0,5710^∗^	+0,5875^∗∗^	+0,7052^∗∗^	+0,5684^∗∗^

*^∗^*p* < 0.05, ^∗∗^*p* < 0.01, ^∗∗∗^*p* < 0.001.*

## Discussion

Alterations in neuronal-glial and/or glial–glial crosstalk may contribute to glial activation in MS, and changes in either neuronal or glial cells could trigger this process. In turn, the resulting functional phenotype will determine the beneficial or detrimental role of microglial cells. Various inhibitory mechanisms, involving both soluble signals and ligand-receptor pairs, coexist to control the pro-inflammatory response of microglial cells under physiological conditions. The presence of glial activation in MS suggests that these mechanisms have been overloaded, implicating them as potential therapeutic targets. In the present study of the dynamics of CD200 and CD200R1 expression in the mouse CNS of an EAE model, we show that changes in the inhibitory CD200-CD200R1 system are already observed at presymptomatic EAE stages, in association with glial activation, with these changes preceding the inflammatory response that accompanies the onset of clinical signs.

The interaction between CD200 and the microglial inhibitory receptor CD200R1 is postulated to be a mechanism involved in controlling the microglial inflammatory response in healthy brain tissue (Tian et al., 2009; Jurgens and Johnson, 2012; Kierdorf and Prinz, 2013). However, CD200 and CD200R1 expression were altered at presymptomatic and symptomatic phases in the CNS of mice that developed EAE, suggesting compromised control of the microglial inflammatory response from early pathological stages. Regarding the CD200 ligand, we looked at the expression of the two Cd200 mRNA variants, Cd200full and Cd200tr, in the mouse brain and spinal cord. Cd200tr mRNA encodes a protein lacking part of the N-terminal domain that is critical for CD200-CD200R1 interaction and CD200R1 stimulation (Hatherley and Barclay, 2004), so acts as an endogenous antagonist of CD200R1 (Chen et al., 2008, 2010). Consequently, the ratio of Cd200full to Cd200tr expression in a tissue may regulate the CD200-CD200R1 interaction. Cd200full mRNA expression was clearly reduced in the spinal cords of MOG-EAE mice during EAE, even during presymptomatic stage, while Cd200tr mRNA expression increased during the symptomatic phase. These changes may decrease the ratio of CD200full to CD200tr, reducing the inhibitory input to microglial/macrophage cells. A sustained decrease in CD200 protein levels was also detected after onset of EAE in the spinal cord. Cd200full mRNA and CD200 protein expression were localized in the cell bodies of motor neurons in the ventral gray matter by *in situ* hybridization and immunohistochemistry, respectively. Interestingly, no CD200 expression was detected in the dorsal gray matter, where interneurons are located. In brain areas, mRNA expression of both CD200 isoforms were altered, but to a lesser extent than in the spinal cord. Curiously, though, there was a clear increase in Cd200tr mRNA expression in brain areas before EAE onset. Together, these results show that the immune reaction induced by MOG_35-55_ administration changed the expression of CD200, suggesting that early motor neuron dysfunction promotes the inflammatory response.

Stimulation of CD200R1 by CD200 activates a signal transduction pathway that inhibits pro-inflammatory gene expression (Zhang et al., 2004). The presence of altered CD200 expression in EAE therefore suggests that mechanisms controlling the inflammatory response are compromised. Previous studies have shown that the manipulation of CD200 expression or function modifies the development of EAE. In this sense, EAE is more severe in CD200-/- than in wild-type mice (Hoek et al., 2000) and the clinical signs of EAE are attenuated in mice that overexpress CD200 (Chitnis et al., 2007). In addition, mice administered anti-CD200 blocking antibody develop a more severe EAE (Meuth et al., 2008). Thus, it is plausible that CD200 levels influence the course of pathology in EAE, and the decreased CD200 expression we observed in MOG-EAE mice, which was already detected at the presymptomatic phase, will probably facilitate the development of pathology. However, it must also be true that the remaining CD200 expression retains a significant functional effect because EAE is more severe when CD200 is completely absent (Hoek et al., 2000) or blocked (Meuth et al., 2008).

Regarding CD200R1, the spinal cords of MOG-EAE mice showed a strong induction of Cd200r1 mRNA expression after the onset of EAE, and this remained elevated during EAE. Cd200r1 mRNA expression also increased in the brain after EAE induction, though to a lesser extent than in the spinal cord. Accordingly, while CD200R1 protein levels were very low in the CNS of control mice, they were markedly increased in the spinal cords of MOG-EAE mice. We clearly localized the CD200R1 mRNA and immunosignal in an increased population of Iba1-positive microglia/macrophages to parenchyma in the dorsal spinal cord white matter of MOG-EAE mice, in and around the areas of demyelination and infiltration. Liu et al. (2010) showed that giving a CD200R1 agonist reduced disease severity, demyelination, and axonal damage in a mouse EAE model, suggesting that CD200R1 stimulation and the resulting inhibition of the inflammatory response positively affect the outcome of EAE. The increase in CD200R1 expression observed in EAE may, therefore, act to compensate for the loss of function in the CD200-CD200R1 system that follows decreased CD200 expression.

CD200R1 agonist treatment has been shown to inhibit the production of pro-inflammatory cytokines in activated glial cells in culture (Liu et al., 2010; Hernangómez et al., 2012; Lyons et al., 2012). Therefore, the increased CD200R1 expression observed in the MOG-EAE mice in our model could be a compensatory response to limit the expression of pro-inflammatory markers. To asses this, we correlated the time course of changes in Cd200 and Cd200r1 mRNA expression in the CNS of MOG-EAE mice with the evolution of the inflammatory response during EAE, and evaluated the RNA expressions of pro-inflammatory M1 (Nos2, Il1b, Tnfa) and anti-inflammatory M2 (Arg1, Il10, Mrc1, Socs3, and Tgfb) markers. In spinal cord regions, the mRNA expression of pro- and anti-inflammatory genes was strongly induced after EAE onset, but only anti-inflammatory gene expression remained elevated thereafter (except for Arg1). A decrease in Cd200full mRNA expression in the spinal cord preceded the inflammatory response, which was attenuated in the presence of a maintained increase in Cd200r1 mRNA expression. However, an inflammatory response also developed in brain areas, though they did not show overall alterations in Cd200full mRNA and Cd200R1 mRNA as in the spinal cord. Curiously, we observed a significant induction of anti-inflammatory genes in brain areas during the presymptomatic phase. The expression pattern of COX2, which is classified as a marker of M1 and M2 phenotypes (David and Kroner, 2011; Chhor et al., 2013; Franco and Fernández-Suárez, 2015), has shown a similar expression pattern to the M2 markers in our MOG-EAE mice. A sustained increase in the expressions of CD200R1 and anti-inflammatory markers could reflect an attempted compensatory response aimed at resolving the inflammatory process (Colton, 2009).

Regarding the clinical score for EAE, a negative correlation was noted with Cd200full mRNA expression in thoracic, cervical but not lumbar spinal cord areas in symptomatic MOG-EAE mice, while a positive correlation was found with Cd200r1 mRNA expression in all spinal cord areas. In addition, a positive correlation was also observed between the clinical score for EAE and Cd200tr mRNA expression in cervical spinal cord. These results suggest that the magnitude of changes in CD200R1 expression in spinal cord reflects the severity of EAE. The magnitude of changes in the mRNA expression of both pro- and anti-inflammatory genes also positively correlated to the severity of EAE spinal cord (mainly lumbar) and brain (mainly rhombencephalon and mesencephalon/diencephalon), and suggests the coexistence of M1 and M2 phenotypes in microglia/macrophages in EAE.

## Conclusion

Our results show that the expressions of CD200 and CD200R1 in the CNS are modified during EAE. *Postmortem* samples from patients with MS are usually only able to show the final stages of disease, and cannot illuminate the changes that occur early in the pathogenesis. In this study, we demonstrated that there was a decrease in CD200 expression before the onset of clinical symptoms in EAE. This suggests that alterations in CD200 expression might also occur in the early stages of MS, which may be responsible for downregulated control of microglial/macrophage activation, thereby stimulating the inflammatory response and contributing to the development of the pathology. By contrast, there was a subsequent increase in CD200R1 expression that possibly represented a compensatory response to re-establish control of the inflammation. The fact that CD200R1 expression was increased points to this receptor as a potential therapeutic target for the regulation of neuroinflammation. Indeed, we consider that CD200R1 agonists are promising molecules that should be developed to modulate neuroinflammation and the resulting neurotoxicity in neurological disease.

## Author Contributions

Conceived and designed the experiments: TV, JSa, and CS. Performed the experiments: TV, JSe, UP, JSa, and CS. Analyzed the data: TV, JSe, JSa, and CS. Wrote the paper: TV and CS. All authors critically revised and approved the final manuscript.

## Conflict of Interest Statement

The authors declare that the research was conducted in the absence of any commercial or financial relationships that could be construed as a potential conflict of interest. The reviewers DT, EJD and the handling Editor declared their shared affiliation, and the handling Editor states that the process nevertheless met the standards of a fair and objective review.
